# Does change in barometric pressure per given time at high altitude influence symptoms of acute mountain sickness on Mount Fuji? A pilot study

**DOI:** 10.1186/s40101-021-00256-y

**Published:** 2021-05-07

**Authors:** Masahiro Horiuchi, Misato Watanabe, Satomi Mitsui, Tadashi Uno

**Affiliations:** Division of Human Environmental Science, Mount Fuji Research Institute, Kami-yoshida 5597-1, Fujiyoshida, Yamanahsi 4030005 Japan

**Keywords:** Arterial hypoxemia, Headache, Perceived sleep quality index, Rapid ascent, Sleep disturbance

## Abstract

**Background:**

Acute mountain sickness (AMS) is a common, transient condition characterized primarily by headaches, and it can also be associated with fatigue, dizziness, and nausea with vomiting. The symptoms of AMS are most pronounced after the first night spent at a new altitude. At sea level, changes in barometric pressure per given time have been associated with migraine headaches. We sought to investigate whether changes in barometric pressure, subjective sleep quality index, and other candidates contributed to the risk of developing AMS on Mount Fuji in Japan.

**Method:**

We surveyed 353 trekkers who stayed overnight at a mountain lodge before summitting Mount Fuji. We collected information regarding sex, age, sleeping altitude at the hut, and perceived sleep quality index including sleep time. AMS was assessed with the Lake Louise Scoring system. Barometric pressure and ambient temperature were collected at the 5th station (2305 m) and at the summit (3776 m).

**Result:**

The overall prevalence of AMS in our cohort was 41.4% (Lake Louise Score ≥ 3 with headache, *n*=146). Using logistic regression, three factors were combined to generate a robust model for determining the risk of AMS (with or without AMS). These included (1) Δ barometric pressure during ascent per hour, (2) sleepiness on rising, and (3) sleep refreshment assessed by perceived sleep quality index.

**Conclusion:**

These results suggest that climbers who stay overnight at the lodge should keep a better physical condition of sleep, and would pay attention to information of barometric pressure condition to decrease their risk of AMS at the summit of Mount Fuji. Our observatory data indicated that an overnight staying in half way up to the summit does not necessarily reduce the AMS risk in both sexes and irrespective of age, at least, until 3776 m elevation.

**Supplementary Information:**

The online version contains supplementary material available at 10.1186/s40101-021-00256-y.

## Background

At high altitudes, barometric pressure (*P*_*B*_) decreases, leading to arterial hypoxemia in human body, which has been considered as the main cause of acute mountain sickness (AMS) [1]. The symptoms of AMS are most pronounced after the first night spent at a new altitude [2]. Indeed, trekkers who stayed overnight at a mountain lodge and had a subjective sleep disturbance were more likely to experience AMS than those who did not, on Mount Fuji in Japan, which was registered as the World Heritage in 2013 [3]. Previous findings using polysomnography demonstrated that impaired sleep efficacy was observed in participants with AMS [4, 5], while AMS was improved without unchanged in sleep efficacy [6]. Additionally, participants did not have AMS despite with an impairment of apnea-hypopnea index [7]. Since sleep has been suggested to be dependent on various factors, such as temperature, wind, hut noise, or smells, comparisons in identical setting are essential [8].

We recently found a positive association between severity of AMS and the magnitude of heart rate decreases that occur with decreasing *P*_*B*_ [9]. Changes to *P*_*B*_ per given time have been associated with migraine headaches at sea level [10–12]. As headache is characterized as the main symptoms of AMS, larger *P*_*B*_ changes at high altitude may be associated with a severity of AMS.

Given these backgrounds, people who will stay overnight at high altitude during mountain trekking would have more likely to experience of AMS under the condition with larger *P*_*B*_ changes. As more than 250,000 people climb on Mount Fuji, and the World Heritage site expects to increase the number of climbers, prevention of AMS at this site is a key concern [3].

We aimed to investigate how changes in *P*_*B*_ contributed to AMS in people who stayed overnight at a lodge on Mount Fuji in Japan. We hypothesized that greater changes in *P*_*B*_ per given time and sleep disturbance were associated with AMS.

## Methods

### Survey site and participants

The survey site was at the 5th station (2305 m) on Mount Fuji, and the date was on August 2019. We asked the participants who begun their descent from the summit of Mount Fuji. We confirmed that all participants stayed one night at a mountain lodge on the ascending road. After providing a detailed explanation of the study, informed consent was obtained from each participant. The study was approved by the ethical committee of Mount Fuji Research Institute in Japan, in accordance with the Declaration of Helsinki.

### Weather conditions

We evaluated *P*_*B*_ at the 5th station (start, 2305 m) and the summit of Mount Fuji (goal, 3776 m) using data from the Japan Meteorological Agency. As secondary variables, ambient temperature (*T*_*a*_) at the 5th station and the summit, and relative humidity at only the summit (due to device limitations) of Mount Fuji.

### Questionnaires

The questionnaires included the following information: “AMS score using the Lake Louise Scoring system (LLS),” “sex,” “age,” “departure and arrival time at the 5th station, and arrival time at the summit of Mount Fuji,” and “sleeping altitude (hut location).” In addition, sleep disturbance was evaluated by perceived sleep quality index by Oguri–Shirakawa–Azumi sleep inventory that was previously used for Japanese populations on Mount Fuji (see below in detail) [13]. This was because revised LLS in 2018 to evaluate AMS excluded a subscale of sleep quality [14]. We also asked their medication status.

### Detection of AMS

We defined AMS as the presence of headache and at least one of the following symptoms: gastrointestinal upset (i.e., anorexia, nausea, or vomiting), fatigue or weakness, dizziness, or lightheadedness with total score ≥ 3 [14]. Although a potential relationship between sleep disturbance and headache, long considered a hallmark of AMS cannot be ignored, sleep disturbance was absent in 40% of cases with severe headache, existing doubts as to whether sleep disturbance was a symptom of AMS [14].

### Perceived sleep quality index

All participants responded to sleeping time and 16 items (each item consists of four choices), and these items were categorized into following 5 subscales: “Sleepiness on rising,” “Initiation and maintenance of sleep,” “Frequency,” “Refreshment,” and “Length of sleep.” Lower scores indicate worse perceived sleep quality [13]. Supplemental Table 1 represents all questionnaire items.

### Statistical analysis

In the univariate analysis of those with and without AMS, a chi-squared test was used to compare categorical variables (sex), and an unpaired *t* test was used to compare continuous variables. Odds ratio of sex was calculated, and 95% confidential interval and effect size were calculated. We determined Cohen’s *d* test for continuous variables and Cramer’s *V* test for categorical variables. We performed multiple logistic regression analysis to detect the factors most associated with AMS (absence/presence; response variables; scored as “0” or “1”). Explanatory variables were as follows: (i) sex (women=0, men=1), (ii) age, (iii–iv) Δ*P*_*B*_ and Δ*T*_*a*_ during ascent per hour (subtracting *P*_*B*_ or *T*_*a*_ values at the 5th station from the summit of Mount Fuji, devising by an individual ascending time), (v) relative humidity at the summit, (vi) sleeping altitude (hut altitude), (vii) sleeping time, and (viii–xii) perceived sleep quality index with the five subscales above mentioned. Although high variance inflation factors (VIFs, > 5) indicates multicollinearity [15], the VIFs were < 4 for all explanatory variables. To find the optimal model containing the parameters that best explain the data, we performed model selection by backward stepwise elimination using Akaike Information Criterion (AIC) based on our previous study [16]. A *P* value less than 0.05 was defined as statistically significant. Statistical analysis was performed using the free software R version 3.1.3.

## Results

While 449 participants completed the surveys, however, 96 were excluded participants from further analysis because of missing information or the presence of acetazolamide, dexamethasone, or analgesics, which may influence AMS symptoms. Thus, we obtained the 353 valid response (78.6% of valid response rate). Overall prevalence of AMS was 41.4 % (*n*=146).

The attributes of the participants and results of univariate analysis are summarized in Table 1. The number of participants who had with or without AMS was calculated for each of the categorical variables (sex), while for the remaining continuous variables the averages and standard deviations were calculated for each category. The optimal model of the multiple logistic regression either for AMS with headache, included three explanatory variables: (1) Δ*P*_*B*_ during ascent, (2) sleepiness on rising, and (3) refreshing (Table 2). For AMS category, partial regression coefficients of “Sleepiness on rising” and “Refreshing” were significant (*P* < 0.05, respectively), whereas that of “Δ*P*_*B*_ per hour from the 5th to the summit” was marginally significant (*P* = 0.097).

**Table 1:** Characteristics of the surveyed independent variables

Independent variables	With AMS	Without AMS	OR	95%CI		Effect size	***P*** values
				Lower	Upper		
(i)*Sex*							
Men	91 (42%)	128 (58%)	1.02	0.66	1.58	0.01	1.000
Women	55 (41%)	79 (59%)					
(ii) Age, years	36.2 ± 13.7	36.0 ± 13.7	-	-3.09	2.72	-0.01	0.903
ΔP_B_ from the 5^th^ to the summit, hPa	-134.5 ± 0.4	-132.7 ± 0.4	-	1.80	1.96	4.84	< 0.001
ΔT_a_ from the 5^th^ to the summit, °C	-10.8 ± 0.9	-10.7 ± 1.0	-	-0.13	0.29	0.09	0.435
Time from the 5^th^ to the summit, min	939 ± 130	971 ± 167	-	-0.11	65.0	0.21	0.051
(iii) ΔP_B_ per hour from the 5^th^ station to the summit, hPa h^-1^	-8.8 ± 1.3	-8.4 ± 1.4	-	0.06	0.65	0.25	0.019
(iv) ΔT_a_ per hour from the 5^th^ station to the summit, °C	-0.71 ± 0.13	-0.68 ± 0.13	-	-0.004	0.05	0.18	0.094
(v) Relative humidity at the summit, %	68.3 ± 17.1	71.7 ± 19.6	-	-0.57	7.34	0.18	0.093
(vi) Hut altitude, m	3195 ± 239	3206 ± 244	-	-40.8	62.0	0.04	0.686
*Perceived sleep quality index*							
(vii) Sleep time, min	192 ± 117	229 ± 115	-	11.9	61.1	0.32	0.004
(viii) Sleepiness on rising	15.8 ± 6.5	19.4 ± 7.1	-	2.10	5.03	0.52	< 0.001
(ix) Initiation, and maintenance of sleep	8.4 ± 7.0	11.3 ± 8.1	-	1.32	4.57	0.39	< 0.001
(x) Frequent dreaming	24.4 ± 8.0	24.1 ± 8.2	-	-2.02	1.43	-0.04	0.736
(xi) Refreshing	9.8 ± 6.7	14.2 ± 7.6	-	2.85	5.93	0.61	< 0.001
(xii) Length of sleep	12.4 ± 7.8	15.1 ± 7.3		1.11	4.31	0.36	< 0.001

For the categorical variable (i.e., sex), the number of participants belonging to different categories and their proportions (in parentheses) are shown. When analyzing, set the top category to 0 and the bottom to 1. For continuous variables (all the others), values are represented as the means ± standard deviations. *P*_*B*_ barometric pressure, *T*_*a*_ ambient temperature, *OR* odds ratio, *CI* confidential interval; - indicates not appreciable. Note that items identified by roman numbers were used for multiple logistic analysis. Items without roman numbers were excluded as these were covariate variables. Note that Cramer’s V-test was determined for “sex” and Cohen’s d-test was determined for other variables

**Table 2:** Summary of the optimal model of multiple logistic regression for “with or without acute mountain sickness (AMS)”

Independent variables	Partial regression coefficient	SE	Odds ratio	95% CI		***P*** values
				Lower	Upper	
ΔP_B_ per hour from the 5^th^ to the summit	-0.137	0.083	0.872	0.741	1.025	0.097
Sleepiness on rising	-0.047	0.018	0.954	0.904	0.968	0.001
Refreshing	-0.067	0.018	0.935	0.920	0.989	<0.001

*SE* standard error; *CI* indicates confidence interval of odds ratio

## Discussion

Previous studies have demonstrated that faster decreases in *P*_*B*_ exacerbate headache [11] and increases in *P*_*B*_ reduce migraine [10]. Although our study was conducted at high altitude and encompassed a larger magnitude of pressure changes compared to the previous studies, our results may be supported with those observations [10, 11], leading to a potential relationship between *P*_*B*_ changes and AMS prevalence. Animal models suggested possible mechanisms to explain *P*_*B*_ change-induced headache is associated with the sympathetic nerve [17], perivascular trigeminal nerve [18], and spinal nerve [19]. Additionally, a previous study found that patients with migraine headaches often claim symptoms of dizziness, which is included in symptoms of AMS [20]. How these animal studies relate to the development of AMS in humans is unclear.

With respect to sleep disturbance, the present results showed that participants with AMS reported lower perceived sleep quality index, though the evaluations were based on subjective feelings. Since previous studies with a direct assessment of sleep disturbance using polysomnography showed equivocal results [4–7], future studies are required. Additionally, participants might have had different symptoms at different time points (e.g., only headache at one point, but dizziness without headache at another point). Although we carefully explained the definition of AMS and asked participants to respond based on the AMS criteria, recall bias might have affected our data. A causal relationship among *P*_*B*_, sleep disturbance, and AMS should be investigated in the future.

As described above, hypoxemia *per se* is one of the main determinant factors to cause AMS [1], and hence, this physiological response cannot be ignored, especially, from the viewpoint of sleep disturbance. This is because peripheral arterial oxygen saturation during sleep was markedly lower, and it did not recover at the onset of rising in the morning [13]. Together, subjective sleep quality index (i.e., sleepiness on rising and refreshing), which was derived as related variables with AMS, could be possibility affected by hypoxemia. Additionally, since the altitude of departure is 2305 m, the participants were exposed to hypobaric hypoxia for more than 15 h (Table 1). Respiratory alkalosis due to increased ventilatory response through an enhancement of chemoreceptor activations necessarily occurs during staying at that environment [4]. However, reduced PCO_2_ caused by respiratory alkalosis inhibits respiratory responses during sleeping. This opposed respiratory sequence further enhances sleeping disturbance and hypoxemia, resulting in a cerebrovascular vasodilation that is likely to induce a headache. We could not conduct in vivo measurements in a large number of participants at this natural environment, however, our observatory data indicated that an overnight staying in half way up to the summit does not necessarily reduce the AMS risk in both sexes and irrespective of age, at least, until 3776 m elevation. Rather, sleeping condition influenced the AMS. Study limitations should also be considered for the future studies, because LLS does not consider other effects, such as smoking [21] and alcohol intake [22], on the AMS risk.

## Conclusion

There were no effects of sex and age on symptoms of AMS. We have associated sleepiness on rising and a lack of refreshment sleep with risk of developing AMS. The optimal model of the multiple logistic regression also included Δ*P*_*B*_ per hour during ascent. These results suggest that climbers who stay overnight at the lodge should keep a better sleep condition, and would pay attention to information of barometric pressure condition to decrease their risk of AMS at the summit of Mount Fuji.

## Supplementary Information

**Additional file 1: Table S1**. Questionnaire used in the present study.

## Data Availability

All data generated or analyzed during this study are included in this published article.

## Abbreviations

*P*_*B*_: Barometric pressure
AMS: Acute mountain sickness
*T*_*a*_: Ambient temperature
LLS: Lake Louise Scoring system
VIF: Variance inflation factors
AIC: Akaike Information Criterion
