# Supplementary material for: Does change in barometric pressure per given time at high altitude influence symptoms of acute mountain sickness on Mount Fuji? A pilot study
Source: J Physiol Anthropol. 2021 May 7;40:6. doi: 10.1186/s40101-021-00256-y (PMC8105930; doi:10.1186/s40101-021-00256-y)
Supplement: Supplementary file 1 — Additional file 1: Table S1. Questionnaire used in the present study. [file 40101_2021_256_MOESM1_ESM.docx]

**Supplemental table 1. Questionnaire used in the present study.**

| **Please respond to following questions.** | | |
| --- | --- | --- |
| Age | years old | |
| Sex | 1. Male | 2. Female |
| Departure time at the 5^th^ station | : | |
| Arrival time at the top | : | |
| Overnight stay location | Hut name: | |
| Please indicate any medications that you are taking or took before trekking. Especially for any cardio- or cerebro-vascular diseases, and any effects on acute mountain sickness, such as acetazolamide, dexamethasone, or analgesics. | Medications: | |

| **Please tell me about your symptoms of acute mountain sickness a recalling the worst condition. Please mark only one appropriate answer with a circle** | | |
| --- | --- | --- |
| Headache | 0. No headache | 1. Mild headache |
|  | 2. Moderate headache | 3. Severe incapacitating headache |
| Gastrointestinal symptoms | 0. No gastrointestinal symptoms | 1. Poor appetite or nausea |
|  | 2. Moderate nausea or vomiting | 3. Severe incapacitating nausea or vomiting |
| Fatigue and/or weakness | 0. No tired or weakness | 1. Mild fatigue/weakness |
|  | 2.Moderate fatigue/weakness | 3. Severe incapacitating fatigue/weakness |
| Dizziness/lightheadedness | 0. No dizzy | 1. Mild dizziness |
|  | 2. Moderate dizziness | 3. Severe incapacitating dizziness |

**Please answer following questions with remembering from last night to this morning.**

a. Time when went to bed last night: (AM･PM)　 　:

b. Time when woke up this morning: (AM･PM)　 　:

c. Sleep hours：About　　　h　　　min

| Based on your sleeping condition through the last night and current physical and mental condition, please mark only one appropriate answer with a circle. | | | | | |
| --- | --- | --- | --- | --- | --- |
|  | Very much | Somewhat | Somewhat | Very much |  |
| 1. Feel tired | 1 | 2 | 3 | 4 | Feel restorative |
| 2. Power concentration | 1 | 2 | 3 | 4 | Poor oncentration |
| 3. Good sleep | 1 | 2 | 3 | 4 | Bad sleep |
| 4. Feeling of freedom | 1 | 2 | 3 | 4 | Under stress |
| 5. Feel run-down | 1 | 2 | 3 | 4 | Shapeup |
| 6. Good appetite | 1 | 2 | 3 | 4 | Poor appetite |
| 7. Dose off till asleep | 1 | 2 | 3 | 4 | Not dose off till asleep |
| 8. Clear my mind | 1 | 2 | 3 | 4 | Daydreaming or feeling dazed |
| 9. Lots pf nightmare | 1 | 2 | 3 | 4 | Without nightmare |
| 10. Fell asleep well | 1 | 2 | 3 | 4 | Fell asleep bad |
| 11. Uncomfortable feeling | 1 | 2 | 3 | 4 | Renewed feeling |
| 12. Dream many times | 1 | 2 | 3 | 4 | No dream |
| 13. Awoke very often during sleep | 1 | 2 | 3 | 4 | No awoke during sleep |
| 14. Can answer question immediately | 1 | 2 | 3 | 4 | Too much trouble to answer |
| 15. Long sleep hours | 1 | 2 | 3 | 4 | Short sleep hours |
| 16. Slept lightly | 1 | 2 | 3 | 4 | Slept soundly |

The questions are over. We appreciate your cooperation.
